# Frequency and Characterization of Antimicrobial Resistance and Virulence Genes of Coagulase-Negative Staphylococci from Wild Birds in Spain. Detection of *tst*-Carrying *S. sciuri* Isolates

**DOI:** 10.3390/microorganisms8091317

**Published:** 2020-08-29

**Authors:** Laura Ruiz-Ripa, Paula Gómez, Carla Andrea Alonso, María Cruz Camacho, Yolanda Ramiro, Javier de la Puente, Rosa Fernández-Fernández, Miguel Ángel Quevedo, Juan Manuel Blanco, Gerardo Báguena, Myriam Zarazaga, Ursula Höfle, Carmen Torres

**Affiliations:** 1 Área de Bioquímica y Biología Molecular, Universidad de La Rioja, 26006 Logroño, Spain; laura_ruiz_10@hotmail.com (L.R.-R.); paula_gv83@hotmail.com (P.G.); rosa.fernandez.1995@gmail.com (R.F.-F.); myriam.zarazaga@unirioja.es (M.Z.); 2 Servicio de Microbiología, Hospital San Pedro, 26006 Logroño, Spain; caalonso@riojasalud.es; 3 Grupo SaBio, Instituto de Investigación en Recursos Cinegéticos IREC (CSIC-UCLM-JCCM), 13005 Ciudad Real, Spain; mcruzceresco@gmail.com (M.C.C.); faunicalatina.vet@gmail.com (Y.R.); ursula.hofle@uclm.es (U.H.); 4 SEO/BirdLife, Citizen Science Unit, 28053 Madrid, Spain; jdelapuente@seo.org; 5 Parque Nacional de la Sierra de Guadarrama, Centro de Investigación, Seguimiento y Evaluación, 28740 Rascafría, Spain; 6 Zoobotánico Jerez, 11408 Jerez de la Frontera, Spain; miguel.quevedo@aytojerez.es; 7 Aquila Foundation, 45567 Oropesa, Spain; aquila.foundation@hotmail.com; 8 Fundación para la Conservación del Quebrantahuesos, 50001 Zaragoza, Spain; gerardo@quebrantahuesos.org

**Keywords:** coagulase-negative staphylococci, CoNS, MRCoNS, wild birds, *tst*, *S. sciuri*, *S. lentus*, antibiotic resistance, reservoir

## Abstract

The objective of this study was to determine the prevalence and diversity of coagulase-negative staphylococci (CoNS) species from wild birds in Spain, as well as to analyze the antimicrobial resistance phenotype/genotype and the virulence gene content. During 2015–2016, tracheal samples of 242 wild birds were collected in different regions of Spain for staphylococci recovery. The species identification was performed using MALDI-TOF. The antimicrobial resistance phenotype and genotype was investigated by the disk diffusion method and by PCR, respectively. The presence of the virulence genes *lukF*/*S*-PV, *tst*, *eta*, *etb*, *etd* and *scn* was investigated by PCR. Moreover, CoNS carrying the *mecA* gene were subjected to SCC*mec* typing. Of the tested animals, 60% were CoNS-carriers, and 173 CoNS isolates were recovered from the 146 positive animals, which belonged to 11 species, with predominance of *S. sciuri* (*n* = 118) and *S. lentus* (*n* = 25). A total of 34% of CoNS isolates showed a multidrug resistance phenotype, and 42 *mecA*-positive methicillin-resistant CoNS (MRCoNS) were detected. The isolates showed resistance to the following antimicrobials (percentage of resistant isolates/antimicrobial resistance genes detected): penicillin (49/ *blaZ*, *mecA*), cefoxitin (24/ *mecA*), erythromycin and/or clindamycin (92/ *erm*(B), *erm*(C), *erm*(43), *msr*(A), *mph*(C), *lnu*(A), *lsa*(B), *vga*(A) and *sal*(A)), gentamicin and/or tobramycin (5/ *aac*(6′)-Ie-*aph*(2″)-Ia, *ant*(4′)-Ia), streptomycin (12/*str*), tetracycline (17/ *tet*(K), *tet*(L), *tet*(M)), ciprofloxacin (4), chloramphenicol (1/ *fexA*), fusidic acid (86/ *fusB*, *fusD*) and trimethoprim–sulfamethoxazole (1/ *dfrK*). None of the isolates harbored the *lukF*/*S*-PV, *eta*, *etb*, *etd* and *scn* genes, but two *S. sciuri* isolates (1%) carried the *tst* gene. Wild birds are frequently colonized by CoNS species, especially *S. sciuri*. We identified scavenging on intensively produced livestock and feeding on landfills as risk factors for CoNS carriage. High proportions of MRCoNS and multidrug resistant CoNS were detected, which coupled with the presence of important virulence genes is of concern.

## 1. Introduction

Staphylococci can be divided in two major groups based on their capacity to produce the enzyme coagulase and, hence, their ability to clot the blood plasma: coagulase-positive (CoPS) and coagulase-negative staphylococci (CoNS). CoNS constitute a very heterogeneous group that comprise over 40 commensal species of the mucous membranes and skin of humans and other animals, especially mammals and birds [1,2,3,4]. However, in the last decade, CoNS have also been recognized as important causative agents of nosocomial infections, especially the species *S. haemolyticus*, *S. epidermidis*, *S. saprophyticus* and *S. lugdunensis* [3,4]. Moreover, CoNS can also cause disease in animals [1,5], including dermatitis and endocarditis in poultry [6,7].

Methicillin-resistant CoNS (MRCoNS), mostly mediated by the *mecA* gene, have gained interest in recent years because of their implications in human and veterinary medicine [8]. Besides methicillin resistance, CoNS have been postulated as an important reservoir of antimicrobial resistance genes that are often located on mobile genetic elements, and, therefore, could be transferred to more pathogenic bacteria, such as *S. aureus,* by horizontal gene transfer [3,4,9,10]. In fact, the CoNS belonging to the *Staphylococcus sciuri* group, which includes *S. sciuri*, *S. fleurettii*, *S. lentus*, *S. vitulinus* and *S. stepanovicii*, are especially relevant because of their role in the origin, evolution and spread of the *mecA* gene [4,11]. Regarding the pathogenicity of CoNS, former studies have reported major virulence factors of *S. aureus* (e.g., Panton–Valentine leukocidin, toxic shock syndrome toxin and exfoliative toxins) in CoNS recovered from humans, livestock and the livestock environment, although their detection is still highly unusual [1,2,12,13].

Several studies have investigated the molecular characteristics of CoNS isolated from food items, companion animals, livestock and clinical samples [1,5,8,13,14,15,16,17], but CoNS from wildlife remains largely unattended. In this regard, birds have been postulated as sentinels, reservoirs and potential disseminators of antimicrobial resistance due to their interaction with the human interface, their diverse ecological niches and their capacity to migrate long distances [18]. Thus, the objective of the present study was to evaluate the prevalence and diversity of CoNS species in wild birds, to determine their antimicrobial susceptibility pattern and to analyze their virulence gene content.

## 2. Materials and Methods

### 2.1. Sampling

From May 2015 to July 2016, the tracheal samples of 242 healthy wild birds [cinereous vulture (*Aegypius monachus*), 98; magpie (*Pica pica*), 59; red kite (*Milvus milvus*), 38; northern bald ibis (*Geronticus eremita*), 27; bearded vulture (*Gypaetus barbatus*), 9; black-headed gull (*Chroicocephalus ridibundus*), 6; Egyptian vulture (*Neophron percnopterus*), 2; European honey buzzard (*Pernis apivorus*), 2; and western marsh harrier (*Circus aeruginosus*), 1] were collected in different regions of northern (Huesca), central (Ciudad Real, Madrid, Valencia and Castellón) and southern (Cádiz) Spain.

Most of the species sampled are obligate scavengers (bearded vulture, Egyptian vulture and cinereous vulture) or facultative scavengers that primarily feed on carrion (red kite). Magpies, black headed gulls and western marsh harriers are occasional scavengers, while honey buzzards and northern bald ibis feed primarily on insects (wasps in particular in honey buzzards). All samples, except magpies and black-headed gulls, were obtained as part of conservation programs, either during banding or radio-tagging of chicks in the nest (cinereous vulture, Egyptian vulture, honey buzzard, northern bald ibis and bearded vulture), pre-reintroduction health checks of captive raised chicks (northern bald ibis and bearded vulture), or adults captured at vulture restaurants for banding and radio-tagging (red kites, bearded vultures and western marsh harrier). Magpies were collected from hunting estates after capture and euthanasia as part of authorized control programs, and black-headed gulls were juveniles dead from trauma in a thunderstorm collected near a landfill.

Chicks were sampled at the end of the breeding season, between May and July, specifically: bearded vulture, June 2015 and June 2016; cinereous vultures, June–July 2015 and June–July 2016; northern bald ibis, June 2016; Egyptian vulture, July 2016; and honey buzzard, July 2016. Black-headed gulls were collected in May 2015, and magpies were captured in May–July 2015 and May–July 2016. Two adult bearded vultures were captured for transmitter exchange at a vulture restaurant in November 2015, and migratory or wintering red kites and the western marsh harrier were captured at a different vulture restaurant during the time of Southbound migration/arrival of wintering birds in November 2015 and during spring migration in February 2016.

None of the birds was specifically captured for the purpose of the present study and capture and handling of the birds was authorized in each case by permits from the regional government. Handling and sampling were carried out following all applicable international, national, and/or institutional guidelines for the care and ethical use of animals, specifically directive 2010/63/EU and Spanish laws 9/2003 and 32/2007, and Royal decrees 178/2004, 1201/2005 and RD53/2013.

All samples were maintained at 4 °C until arrival at the laboratory and frozen at −80 °C until further analysis. These samples were tested in parallel for the presence of CoPS [19] and also for CoNS; this last one in the present study. In the previous work, CoPS were recovered from 20 of the 242 samples analyzed (8.3%) and they were identified as *S. aureus* (*n* = 9) and *S. delphini* (*n* = 12) [19].

### 2.2. Bacterial Isolation and Identification

The tracheal swab samples were inoculated into brain heart infusion (BHI; Condalab, Madrid, Spain) broth supplemented with 6.5% NaCl and incubated for 24 h at 37 °C. An aliquot of 30 µL was seeded on mannitol salt agar (MSA; Condalab, Madrid, Spain) and oxacillin resistance screening agar base (ORSAB; Oxoid, Hampshire, UK) with 2 mg/L oxacillin and incubated for 24 h at 37 °C, for CoNS and MRCoNS recovery. A maximum of five mannitol non-fermenting colonies per sample were selected and identified by matrix-assisted laser desorption/ionization time-of-flight (MALDI-TOF; Bruker Daltonics, Bremen, Germany) using the standard extraction protocol recommended by Bruker [20]. Isolates belonging to different CoNS species of each sample were further studied.

### 2.3. Antimicrobial Resistance Phenotype and Genotype

The susceptibility to 13 antimicrobial agents was tested by the disk diffusion method. The antimicrobial agents tested were as follows (µg/disk): penicillin (10 units), cefoxitin (30), erythromycin (15), clindamycin (2), gentamicin (10), tobramycin (10), streptomycin (10), tetracycline (30), ciprofloxacin (5), chloramphenicol (30), linezolid (30), fusidic acid (10) and trimethoprim–sulfamethoxazole (1.25 + 23.75). The disk diffusion results for all antimicrobial agents were interpreted using the European Committee on Antimicrobial Susceptibility Testing (EUCAST) zone diameter breakpoints for CoNS [21] when available, with the exception of streptomycin [22]. As *S. sciuri* is intrinsically resistant to clindamycin, this resistance was not taken into account when considering the isolate as multiresistant [23].

The presence of the following resistance genes was tested by single PCRs: *blaZ*, *mecA*, *mecB*, *mecC*, *erm*(A), *erm*(B), *erm*(C), *erm*(T), *erm*(43), *msr*(A), *mph*(C), *sal*(A), *lnu*(A), *lnu*(B), *lsa*(B), *vga*(A), *aac*(6′)-Ie-*aph*(2″)-Ia, *ant*(4′)-Ia, *str*, *ant*(6), *tet*(L), *tet*(M), *tet*(K), *fex*(A), *fex*(B), *cat*_pC194_, *cat*_pC221_, *cat*_pC223_, *cfr*, *cfr*(B), *cfr*(D), *optrA*, *poxtA*, *fusB*, *fusC*, *fusD*, *dfr*A, *dfrD*, *dfrG* and *dfrK* (Table S1). The physical linkage of *tet*(L)-*dfrK* was investigated by PCR (Table S1). Positive controls from the collection of the Universidad de La Rioja were included in all PCR assays. In addition, mutations leading to amino acid changes in the GyrA and GrlA proteins were investigated in ciprofloxacin-resistant isolates by PCR and amplicon sequencing (Table S1). The respective sequences of *S. lentus* strain NCTC12102 (GenBank accession number UHDR01000002), *S. sciuri* strain NCTC12103 (GenBank accession number LS483305) and *S. xylosus* strain NCTC11043 (GenBank accession number UHEI01000002) were used as reference for the amino acid changes detection.

### 2.4. Staphylococcal Cassette Chromosome mec (SCCmec) Characterization

All CoNS isolates carrying the *mecA* gene were subjected to SCC*mec* typing by multiplex PCRs targeting the *ccr* gene complex and the *mec* gene complex, as previously described [24] (Table S1).

### 2.5. Virulence Gene Content

The presence of the genes encoding the virulence determinants Panton–Valentine leukocidin (*lukF*/*S*-PV), toxic shock syndrome toxin 1 (*tst*), and the exfoliative toxins A (*eta*), B (*etb*) and D (*etd*) was studied by single PCRs and confirmed by amplicon sequencing (Table S1). Moreover, the presence of the *scn* gene, the marker of the human immune evasion cluster (IEC), was investigated in all CoNS isolates (Table S1). Positive controls from the collection of the Universidad de La Rioja were included in all PCR assays.

### 2.6. Statistical Analysis

Pearson’s chi-square test was used to explore colonization of the tested birds by *S. sciuri* and *S. lentus*. Specifically, we compared the carriage of *S. sciuri* and *S. lentus* between obligate or facultative scavengers and species less likely to feed on carrion (especially livestock carrion), and in the case of *S. lentus* carriage, in red kites captured during and on spring (return) migration (February 2016, *n* = 25) and kites arriving at the vulture restaurant on Southbound migration (November 2015, *n*=13). Analyses were carried out using SPSS statistical software version 24.0 (IBM^®^, SPSS Inc., Chicago, IL, USA) and significance was set at *p* ≤ 0.05.

## 3. Results

### 3.1. Occurrence of CoNS and Species Identification

In this study, 146 out of 242 (60.3%) tested wild birds were colonized by, at least, one species of CoNS (Table 1). Among the 146 positive birds, one single CoNS species was detected in 120 of them. Co-carriage of two different species was identified in 24 animals, and co-carriage of three and four different species was detected in 1 animal each. The following patterns of co-carriage of CoNS species were detected among the positive birds (number of animals/animal’s species): *S. sciuri/S. lentus* (12/red kite), *S. sciuri/S. fleurettii* (5/cinereous vulture), *S. sciuri/S. xylosus* (2/cinereous vulture), *S. sciuri/S. kloosii* (2/cinereous vulture and European honey buzzard), *S. epidermidis/S. capitis* (1/magpie), *S. sciuri/S. epidermidis* (1/ northern bald ibis), *S. saprophyticus/ S. kloosii* (1/ European honey buzzard), *S. sciuri/S. fleurettii/S. schleiferi* subsp. *schleiferi* (1/ bearded vulture), and *S. sciuri/S. lentus/S. vitulinus/S. xylosus* (1/ western marsh harrier).

In total, 173 CoNS isolates were recovered, and the MALDI-TOF results revealed the presence of 11 different species (number of isolates recovered/ percentage in relation to the total isolates): *S. sciuri* (118/68.2)*, S. lentus* (25/14.5), *S. fleurettii* (7/4), *S. vitulinus* (6/3.5), *S. epidermidis* (4/2.3), *S. kloosii* (3/1.7), *S. schleiferi* subsp. *schleiferi* (3/1.7), *S. xylosus* (3/1.7), *S. saprophyticus* (2/1.2), *S. succinus* (1/0.6) and *S. capitis* (1/0.6) (Table 1). While *S. sciuri* was present in all species, it was detected significantly more frequently in obligate and facultative scavengers (Pearson’s χ^2^ = 11.34, D.f. = 1, *p* = 0.001, Table 1). In addition, *S. lentus* was only detected in obligate or frequent scavengers and in the harrier that had been captured on the vulture restaurant. Red kites captured on return migration (February 2016) were colonized significantly more frequently by *S. lentus* than those captured on Southbound migration/arrival at the vulture restaurant (November 2015, Pearson’s χ^2^ = 6.89, D.f. = 1, *p* = 0.0087). 

### 3.2. Antimicrobial Resistance Phenotype and Genotype

Table 2 shows the antimicrobial resistance rates detected among the 173 CoNS recovered from wild birds. The single isolate that showed susceptibility to all the antimicrobial agents evaluated was the *S. capitis* isolate. Fifty-eight (34%) multidrug-resistant (MDR) isolates (resistant to at least three different classes of antimicrobial agents) were detected: *S. sciuri* (*n* = 21), *S. lentus* (*n* = 20), *S. fleurettii* (*n* = 6), *S. epidermidis* (*n* = 4), *S. schleiferi* (*n* = 3), *S. xylosus* (*n* = 2), *S. kloosii* (*n* = 1) and *S. vitulinus* (*n* = 1).

Table 3 summarizes the antimicrobial resistance phenotypes and genotypes of CoNS, while the specific characterization of each isolate recovered in this study can be found in the Supplementary Table S2. Forty-two *mecA*-carrying MRCoNS isolates (24%) were detected: *S. sciuri* (*n* = 28), *S. lentus* (*n* = 5), *S. fleurettii* (*n* = 5), *S. vitulinus* (*n* = 2) and *S. epidermidis* (*n* = 2). Moreover, five isolates belonging to the species *S. sciuri* or *S. fleurettii* also carried the *mecA* gene but did not show a methicillin resistance phenotype (Table 3). Eighty-five CoNS showed penicillin resistance that was mediated by the *mecA* and/or the *blaZ* genes in 59 of them. However, the mechanism of penicillin resistance could not be identified in the remaining isolates. Macrolide and/or lincosamide resistance was mediated by different combinations of *erm*(B), *erm*(C), *erm*(43), *msr*(A), *mph*(C), *lnu*(A), *lsa*(B), *vga*(A) and *sal*(A) resistance genes. The *sal*(A) gene was solely found among the *S. sciuri* isolates. The *aac*(6′)-Ie-*aph*(2″)-Ia, *ant*(4′)-Ia and/or *str* genes were detected among the aminoglycoside-resistant isolates. Tetracycline resistance, which was especially high among the *S. lentus* isolates (60%), was mediated by the *tet*(K), *tet*(L) and/or *tet*(M) genes. The analysis of the PCR-amplicon sequencing results revealed the presence of amino acid changes in the GyrA (S84L) and GrlA (D84E and M89L) proteins in five ciprofloxacin-resistant *S. lentus* isolates. Moreover, one *S. sciuri* isolate and one *S. xylosus* isolates were ciprofloxacin-resistant, but no amino acid changes were detected. The *fexA* gene was found in the two *S. sciuri* isolates that showed chloramphenicol resistance. Although only one out of the two *S. saprophyticus* isolates presented phenotypic resistance to fusidic acid, both isolates harbored the *fusD* gene, which is only detected in this species and has been reported to confer intrinsic resistance. The mechanisms implicated in the fusidic acid resistance in the remaining isolates were only identified in one *S. epidermidis* that harbored the *fusB* gene. The *dfrK* gene was identified in the single trimethoprim-resistant isolate detected (Table 3), and the PCR and amplicon sequencing results revealed that it was linked to the *tet*(L) gene. All CoNS exhibited linezolid susceptibility.

### 3.3. Virulence Gene Content

None of the isolates carried the *lukS/F*-PV, *eta*, *etb*, *etd* or *scn* genes. Interestingly, two *S. sciuri* isolates recovered from cinereous vultures carried the *tst* gene (Table 3).

### 3.4. SCCmec Typing

Among the 47 *mecA*-positive CoNS isolates, ten *S. sciuri* and two *S. lentus* isolates were typed as SCC*mec*-III and one *S. epidermidis* as SCC*mec*-IV. No consensus for the SCC*mec* type was determined for the remaining *mecA*-carrying CoNS, either because they were not ascribed to a previously known SCC*mec* type, or because they were non-typeable with the primers used (Table S3).

## 4. Discussion

The current work represents the largest study characterizing CoNS recovered from healthy free-ranging birds, and provides novel information about the frequency, diversity of species, antimicrobial resistance phenotype/genotype and the virulence profile among CoNS from wild animals.

The CoNS tracheal carriage rate detected in wild birds (60%) was higher than that detected in a previous study conducted among wild boars in Spain (37.7%) [25], but similar to that among birds of prey in Portugal (75%) [26]. These results suggest that birds (or at least those consuming vertebrate or invertebrate prey) are more frequently colonized by CoNS than mammals; however, further studies in other animal species need to be assessed to corroborate these data. In this study, a high diversity of CoNS species was detected, *S. sciuri* being the predominant one accounting for nearly 70% of the isolates recovered. This was to be expected since this species is known to has broad host range and is adapted to very different habitats [4,8,12,27]. Moreover, although it has been formerly found causing infections in animals [15], *S. sciuri* is the most common CoNS species colonizing healthy wild animals, including birds [25,26]. *S. lentus* was the second species most frequently recovered in this study and was especially prevalent among red kites. This staphylococcal species is commonly detected among farm animals and people with professional exposure to livestock [16,28,29]. More frequent colonization of obligate and frequent scavengers by *S. sciuri* and *S. lentus* suggests that despite being respiratory tract colonizers, carrion feeding may increase the exposure. In particular, carriage of *S. lentus* by red kites was higher in February than in November when kites arriving from breeding grounds in northern and central Europe were captured. Both the difference in prevalence and the high rate of tetracycline resistance detected in this staphylococcal species provide circumstantial evidences that *S. lentus* may be acquired from livestock carrion, particularly from slaughterhouse remains (pork) and deceased chickens from commercial layer and broiler farms that are employed as food at the vulture restaurant where the birds were captured. The species *S. xylosus* and *S. kloosii* were also recovered from birds of prey in Portugal [26]. Other CoNS species that are frequently detected as causative agents of diverse diseases in humans, such as *S. epidermidis*, *S. saprophyticus*, *S. succinus* and *S. capitis* [4], were also found among healthy wild birds at very low rates.

The antimicrobial resistance rates detected for some antimicrobials among the isolates investigated are of great concern, especially because wild birds are not supposed to be under the selective pressure of antimicrobial agents; this contrasts with the high susceptibility rates detected among CoNS from wild boars in a previous Spanish study [25]. In our work, 24% of the CoNS isolates showed methicillin resistance, which is far higher than the rate observed in mammals (2.5%) and in birds of prey (0%) [25,26]. Methicillin resistance was mediated in all cases by the *mecA* gene, which was not surprising, as *mecC*-carrying MRCoNS isolates are still scarce [30], and, as far as we know, the *mecB* gene has not been previously reported in CoNS species. However, in the previous study that characterized the CoPS isolates of these samples, *mecC*-positive methicillin-resistant *S. aureus* (MRSA) were detected [19]. Moreover, five *S. sciuri* or *S. fleurettii* isolates harbored the *mecA* gene but were phenotypically susceptible to cefoxitin, which has been formerly reported among CoNS of diverse origins [12,31]. *mecA* gene homologues that are not always associated with methicillin resistance have been found in *S. sciuri* and *S. fleurettii* [11], but the primers used in this study do not amplify these *mecA* homologues. Hence, the methicillin-susceptible phenotype could be attributed to the heterogenous expression of the *mecA* gene. We could not identify the mechanism implicated in penicillin resistance in 26 isolates, which has been formerly reported among CoNS [26,27]. This fact suggests that other unknown resistance mechanisms are present in the isolates investigated or that the breakpoints for this antimicrobial are not accurate for CoNS. The extremely high resistance rate to clindamycin detected (90%) is worrisome as this antimicrobial is widely used in clinical and veterinary medicine. Among the *S sciuri* isolates, clindamycin resistance was mainly mediated by the presence of the *sal*(A) gene, which confers intrinsic resistance to lincosamides and streptogramin A antimicrobials in this species [32]. Although the *sal*(A) gene has been previously described in non-*S. sciuri* CoNS species from pets in China [33], in this work, it was only detected in *S. sciuri*. Macrolides and lincosamides resistance mediated by the *erm*(43) gene was detected in seven *S. lentus* isolates. This gene has been formerly detected among isolates belonging to the species *S. lentus* and *S. sciuri* of diverse origins, including healthy wild animals [25,34]. It is necessary to note the high tetracycline resistance rate (60%) observed among the *S. lentus* isolates with respect to the overall tetracycline rate of CoNS (17%), which was mostly mediated by the *tet*(K) gene. Similar resistance rates in *S. lentus* were detected in isolates recovered from livestock [29,35], which could be attributable to the extended use of this antimicrobial in veterinary medicine. The location of *dfr*(K) next to the *tet*(L) gene in one *S. lentus* isolate suggests their location in a pKKS2187-like plasmid previously reported among MRSA-CC398 and MRCoNS of pig origin [29,36]. This supports the hypothesis that red kites could acquire CoNS from livestock carrion at the vulture restaurant via direct contact or the food chain. In cinereous vulture chicks, the potential route of colonization is less obvious. Cinereous vultures generally feed on medium-sized native carrion and, in the case of this colony situated in a National Park close to Madrid, no feeding station (vulture restaurant) is in the range of foraging of the vultures during chick raising. However, observation of birds ringed at the colony foraging on solid urban waste landfills [37] suggests that one of the potential sources for colonization by CoNS could be exposure of the adults in the landfill environment and subsequent colonization of the nestlings, or exposure of the latter from landfill foraged food, as has been shown for CoPs in white stork nestlings [38]. However, to date, no detailed information on the degree of exposure of the cinereous vulture chicks to landfill foraged food is available. Fusidic acid resistance rates above 50% were also reported in CoNS from livestock and the livestock environment and birds of prey [8,9,12,26,29], which suggests the presence of mutations in the *fusA* gene or intrinsic resistance genes like the *fusD* in *S. saprophyticus* and *fusE* in *S. cohnii* subsp. *urealyticus* [39]. Fortunately, all CoNS isolates from wild birds exhibited susceptibility to linezolid, which is considered as a last resort antimicrobial in human medicine used to treat serious infections caused by multidrug resistant Gram-positive bacteria, including MRCoNS.

In this study, two *S. sciuri* isolates harbored the *tst* gene encoding the pyrogenic toxin superantigen TSST-1 that is considered one of the most important virulence factors produced by *S. aureus*. It is located on staphylococcal pathogenicity islands (SaPIs) and its mobilization is assisted by different phages [40]. This gene has been formerly described among clinical CoNS isolates but also among those recovered from bovine milk [2,13,41]; however, to best of our knowledge, this is the first description of *tst*-carrying CoNS isolates from wild animals. Apart from the enterotoxigenic potential, few studies that explore the prevalence of major virulence factors of *S. aureus* in CoNS exist.

As previously reported by other authors, the SCC*mec* type IV and, especially, type III are the most common types detected among CoNS isolates of animal origin [8,28,42]. However, the majority of *mecA*-carrying isolates could not be SCC*mec* typed with the primers used, which highlights the high diversity of SCC*mec* types among CoNS and suggests the presence of novel SCC*mec* elements different from those found in MRSA isolates.

## 5. Conclusions

Free-living predatory birds are frequently colonized by CoNS species, especially *S. sciuri*. In this study we have demonstrated that wild birds are a reservoir of CoNS carrying not only important antimicrobial resistance genes, but also major virulence factors traditionally associated with *S. aureus*. Scavenging on livestock from intensive production (pork and poultry) and foraging on landfills are a potential source of CoNS isolates recovered from wild birds. The detection of two *S. sciuri* isolates carrying the *tst* gene in wildlife is of great concern.

## Figures and Tables

**Table 1:** Number of animals sampled, and diversity of coagulase-negative staphylococci (CoNS) species detected among wild birds.

Animal Species	Number of Animals Sampled	Number of Animals Carrying CoNS (%)	Number of Isolates Recovered	Number of Isolates					
				*Staphylococcus sciuri*	*Staphylococcus lentus*	*Staphylococcus fleurettii*	*Staphylococcus vitulinus*	*Staphylococcus epidermidis*	Other CoNS Species ^1^
**Cinereous vulture**	98	67 (68)	73	55	6	6	1	-	5
**Magpie**	59	23 (39)	24	16	-	-	4	2	2
**Red kite**	38	30 (79)	42	25	17	-	-	-	-
**Northern bald ibis**	27	11 (41)	12	11	-	-	-	1	-
**Bearded vulture**	9	5 (56)	7	2	1	1	-	1	2
**Black-headed gull**	6	5 (83)	5	5	-	-	-	-	-
**Egyptian vulture**	2	2 (100)	2	2	-	-	-	-	-
**European honey Buzzard**	2	2 (100)	4	1	-	-	-	-	3
**Western marsh Harrier**	1	1 (100)	4	1	1	.	1	-	1
**Total**	242	146 (60)	173	118	25	7	6	4	13

^1^ This includes *S. xylosus, S. kloosii, S. schleiferi* subsp. *schleiferi, S. saprophyticus, S. succinus* and *S. capitis.*

**Table 2:** Antimicrobial resistance rates detected among the CoNS isolates recovered from wild birds.

Antimicrobial Resistance for:	PEN	FOX	ERY	CLI	GEN	TOB	STR	TET	CIP	CHL	FUS	SXT
**Percentage of resistant isolates**	49	24	16	92	3	5	12	17	4	1	86	1

PEN, penicillin; FOX, cefoxitin; ERY, erythromycin; CLI, clindamycin; GEN, gentamicin; TOB, tobramycin; STR, streptomycin; TET, tetracycline; CIP, ciprofloxacin; CHL, chloramphenicol; FUS, fusidic acid; SXT, trimethoprim–sulfamethoxazole.

**Table 3:** Antimicrobial resistance phenotype and genotype and virulence gene content in the CoNS recovered from wild birds.

Species	Number of Isolates	Antimicrobial Resistance Phenotype ^1,2^	Antimicrobial Resistance Genotype ^2^	Virulence Gene Content
***S. sciuri***	118	PEN^56^-FOX^28^-ERY^11^-CLI-GEN^3^-TOB^6^-STR^9^-TET^8^-CIP^1^-CHL^2^-FUS^108^	*mecA*^31^, *blaZ*^7^, *erm*(B)^5^, *erm*(C)^8^, *msr*(A)^1^, *lnu*(A)^20^, *lsa*(B)^1^, *sal*(A), *aac*(6′)-Ie-*aph*(2″)-Ia^3^, *ant*(4′)-Ia^3^, *str*^9^, *tet*(K)^6^, *tet*(L)^1^, *tet*(M)^2^, *fexA*^2^	*tst* ^2^
***S. lentus***	25	PEN^7^-FOX^5^-ERY^13^-CLI^24^-TOB^1^-STR^3^-TET^15^-CIP^5^-FUS^21^-SXT^1^	*mecA*^5^, *blaZ*^3^, *erm*(B)^9^, *erm*(C)^4^, *erm*(43)^7^, *mph*(C)^10^, *lnu*(A)^12^, *vga*(A)^2^, *lsa*(B)^1^, *ant*(4′)-Ia^1^, *str*^3^, *tet*(K)^11^, *tet*(L)1, *tet*(M)^5^, *dfrK*^1^	
***S. fleurettii***	7	PEN-FOX^5^-CLI^6^-STR^3^-FUS	*mecA*, *lnu*(A)^1^, *str*^3^	
***S. vitulinus***	6	PEN^2^-FOX^2^-CLI^2^-GEN^1^-TOB^1^-STR^2^-FUS^5^	*mecA*^2^, *aac*(6′)-Ie- *aph*(2″)-Ia^1^, *str*^2^	
***S. epidermidis***	4	PEN-FOX^2^-ERY^3^-CLI-TET^1^-FUS^2^	*mecA*^2^, *blaZ*^3^, *erm*(C)^3^, *mph*(C)^2^, *lnu*(A)^3^, *vga*(A)^1^, *tet*(K)^1^, *fusB*^1^	
***S. kloosii***	3	PEN-CLI^1^-TET^1^-FUS	*blaZ*, *lnu*(A)^1^, *tet*(K)^1^	
***S. schleiferi* subsp. *schleiferi***	3	PEN^1^-CLI^2^-STR-TET	*blaZ*^1^, *lnu*(A)^2^, *str*, *tet*(K)	
***S. xylosus***	3	PEN-ERY^1^-CLI^2^-STR^1^-TET^1^-CIP^1^-FUS^2^	*blaZ*^1^, *erm*(B)^1^, *mph*(C)^1^, *lnu*(A)^1^, *str*^1^, *tet*(K)^1^	
***S. saprophyticus***	2	PEN^1^-GEN^1^-TOB^1^-FUS^1^	*blaZ*^1^, *aac*(6′)-Ie-*aph*(2″)-Ia^1^, *fusD*	
***S. capitis***	1	Susceptible	-	
***S. succinus***	1	PEN	*blaZ*	

^1^ PEN, penicillin; FOX, cefoxitin; ERY, erythromycin; CLI, clindamycin; GEN, gentamicin; TOB, tobramycin; STR, streptomycin; TET, tetracycline; CIP, ciprofloxacin; CHL, chloramphenicol; FUS, fusidic acid; SXT, trimethoprim–sulfamethoxazole.^2^ The superscripts indicate the number of isolates when not all isolates of the group have the same characteristic.

## Supplementary Materials

The following are available online at https://www.mdpi.com/2076-2607/8/9/1317/s1. Table S1: Primer pairs used for the characterization of CoNS recovered from wild birds; Table S2: Antimicrobial resistance phenotype and genotype, and virulence gene content of the 173 CoNS recovered from free-ranging birds; Table S3: SCC*mec* typing of the 47 CoNS isolates from wild birds that carried the *mecA* gene.

## References

[1] Ünal N., Çinar O.D. (2012). Detection of stapylococcal enterotoxin, methicillin-resistant and Panton-Valentine leukocidin genes in coagulase-negative staphylococci isolated from cows and ewes with subclinical mastitis. Trop. Anim. Health Prod..

[2] Bertelloni F., Fratini F., Ebani V.V., Galiero A., Turchi B., Cerri D. (2015). Detection of genes encoding for enterotoxins, TSST-1, and biofilm production in coagulase-negative staphylococci from bovine bulk tank milk. Dairy Sci. Technol..

[3] Heilmann C., Ziebuhr W., Becker K. (2019). Are coagulase-negative staphylococci virulent?. Clin. Microbiol. Infect..

[4] Becker K., Heilmann C., Peters G. (2014). Coagulase-negative staphylococci. Clin. Microbiol. Rev..

[5] Pyörälä S., Taponen S. (2009). Coagulase-negative staphylococci-emerging mastitis pathogens. Vet. Microbiol..

[6] Pyzik E., Marek A., Stȩpień-Pyśniak D., Urban-Chmiel R., Jarosz L.S., Jagiełło-Podȩbska I. (2019). Detection of antibiotic resistance and classical enterotoxin genes in coagulase-negative staphylococci isolated from poultry in Poland. J. Vet. Res..

[7] Stępień-Pyśniak D., Wilczyński J., Marek A., Śmiech A., Kosikowska U., Hauschild T. (2017). *Staphylococcus simulans* associated with endocarditis in broiler chickens. Avian Pathol..

[8] Nemeghaire S., Vanderhaeghen W., Angeles Argudín M., Haesebrouck F., Butaye P. (2014). Characterization of methicillin-resistant *Staphylococcus sciuri* isolates from industrially raised pigs, cattle and broiler chickens. J. Antimicrob. Chemother..

[9] Schoenfelder S.M.K., Dong Y., Feßler A.T., Schwarz S., Schoen C., Köck R., Ziebuhr W. (2017). Antibiotic resistance profiles of coagulase-negative staphylococci in livestock environments. Vet. Microbiol..

[10] Otto M. (2013). Coagulase-negative staphylococci as reservoirs of genes facilitating MRSA infection. BioEssays.

[11] Tsubakishita S., Kuwahara-Arai K., Sasaki T., Hiramatsu K. (2010). Origin and molecular evolution of the determinant of methicillin resistance in staphylococci. Antimicrob. Agents Chemother..

[12] Ruiz-Ripa L., Feßler A.T., Hanke D., Sanz S., Olarte C., Mama O.M., Eichhorn I., Schwarz S., Torres C. (2020). Coagulase-negative staphylococci carrying *cfr* and PVL genes, and MRSA/MSSA-CC398 in the swine farm environment. Vet. Microbiol..

[13] Giormezis N., Kolonitsiou F., Foka A., Drougka E., Liakopoulos A., Makri A., Papanastasiou A.D., Vogiatzi A., Dimitriou G., Marangos M. (2014). Coagulase-negative staphylococcal bloodstream and prosthetic-device-associated infections: The role of biofilm formation and distribution of adhesin and toxin genes. J. Med. Microbiol..

[14] Mama O.M., Morales L., Ruiz-Ripa L., Zarazaga M., Torres C. (2020). High prevalence of multidrug resistant *S. aureus*-CC398 and frequent detection of enterotoxin genes among non-CC398 *S. aureus* from pig-derived food in Spain. Int. J. Food Microbiol..

[15] Chen S., Wang Y., Chen F., Yang H., Gan M., Zheng S.J. (2007). A highly pathogenic strain of *Staphylococcus sciuri* caused fatal exudative epidermitis in piglets. PLoS ONE.

[16] De Martino L.D., Lucido M., Mallardo K., Facello B., Mallardo M., Iovane G., Pagnini U., Tufano M.A., Catalanotti P. (2010). Methicillin-resistant staphylococci isolated from healthy horses and horse personnel in Italy. J. Vet. Diagn. Investig..

[17] Gómez-Sanz E., Ceballos S., Ruiz-Ripa L., Zarazaga M., Torres C. (2019). Clonally diverse methicillin and multidrug resistant coagulase negative staphylococci are ubiquitous and pose transfer ability between pets and their owners. Front. Microbiol..

[18] Bonnedahl J., Järhult J.D. (2014). Antibiotic resistance in wild birds. Upsala J. Med. Sci..

[19] Ruiz-Ripa L., Gómez P., Alonso C.A., Camacho M.C., de la Puente J., Fernández-Fernández R., Ramiro Y., Quevedo M.A., Blanco J.M., Zarazaga M. (2019). Detection of MRSA of Lineages CC130-*mecC* and CC398-*mecA* and *Staphylococcus delphini*-*lnu*(A) in Magpies and Cinereous Vultures in Spain. Microb. Ecol..

[20] Matsuda N., Matsuda M., Notake S., Yokokawa H., Kawamura Y., Hiramatsu K., Kikuchia K. (2012). Evaluation of a simple protein extraction method for species identification of clinically relevant staphylococci by matrix-assisted laser desorption ionization - Time of flight mass spectrometry. J. Clin. Microbiol..

[21] EUCAST European Committee on Antimicrobial Susceptibility Testing (2019). Breakpoint Tables for Interpretation of MICs and Zone Diameters, Version 9.0.

[22] European Society of Clinical Microbiology and Infectious Diseases (2013). Comité de l’antibiogramme de la Société Française de Microbiologie- Recommandation.

[23] Schwarz S., Silley P., Simjee S., Woodford N., van duijkeren E., Johnson A.P., Gaastra W. (2010). Assessing the antimicrobial susceptibility of bacteria obtained from animals. J. Antimicrob. Chemother..

[24] Kondo Y., Ito T., Ma X.X., Watanabe S., Kreiswirth B.N., Etienne J., Hiramatsu K. (2007). Combination of multiplex PCRs for staphylococcal cassette chromosome *mec* type assignment: Rapid identification system for *mec*, *ccr*, and major differences in junkyard regions. Antimicrob. Agents Chemother..

[25] Mama O.M., Ruiz-Ripa L., Lozano C., González-Barrio D., Ruiz-Fons J.F., Torres C. (2019). High diversity of coagulase negative staphylococci species in wild boars, with low antimicrobial resistance rates but detection of relevant resistance genes. Comp. Immunol. Microbiol. Infect. Dis..

[26] Sousa M., Silva N., Igrejas G., Sargo R., Benito D., Gómez P., Lozano C., Manageiro V., Torres C., Caniça M. (2016). Genetic diversity and antibiotic resistance among coagulase-negative staphylococci recovered from birds of prey in Portugal. Microb. Drug Resist..

[27] Gómez P., Casado C., Sáenz Y., Ruiz-Ripa L., Estepa V., Zarazaga M., Torres C. (2017). Diversity of species and antimicrobial resistance determinants of staphylococci in superficial waters in Spain. FEMS Microbiol. Ecol..

[28] Zhang Y., Agidi S., Lejeune J.T. (2009). Diversity of staphylococcal cassette chromosome in coagulase-negative staphylococci from animal sources. J. Appl. Microbiol..

[29] Ugwu C.C., Gomez-Sanz E., Agbo I.C., Torres C., Chah K.F. (2015). Characterization of mannitol-fermenting methicillin-resistant staphylococci isolated from pigs in Nigeria. Braz. J. Microbiol..

[30] Loncaric I., Kübber-heiss A., Posautz A., Ruppitsch W., Lepuschitz S., Schauer B., Feßler A.T., Krametter-frötscher R., Harrison E.M., Holmes M.A. (2019). Characterization of *mecC* gene-carrying coagulase-negative *Staphylococcus* spp. isolated from various animals. Vet. Microbiol..

[31] Kulangara V., Nair N., Sivasailam A., Sasidharan S., Kollannur J.D., Syam R. (2017). Genotypic and phenotypic β-lactam resistance and presence of PVL gene in Staphylococci from dry bovine udder. PLoS ONE.

[32] Hot C., Berthet N., Chesneau O. (2014). Characterization of *sal*(A), a Novel Gene Responsible for Lincosamide and Streptogramin a resistance in *Staphylococcus sciuri*. Antimicrob. Agents Chemother..

[33] Deng F., Wang H., Liao Y., Li J., Feßler A.T., Michael G.B., Schwarz S., Wang Y. (2017). Detection and Genetic Environment of Pleuromutilin-Lincosamide- Streptogramin A Resistance Genes in Staphylococci Isolated from Pets. Front. Microbiol..

[34] Schwendener S., Perreten V. (2012). New MLS B resistance gene *erm*(43) in *Staphylococcus lentus*. Antimicrob. Agents Chemother..

[35] Vanderhaeghen W., Vandendriessche S., Crombé F., Nemeghaire S., Dispas M., Denis O., Hermans K., Haesebrouck F., Butaye P. (2013). Characterization of methicillin-resistant non-*Staphylococcus aureus* staphylococci carriage isolates from different bovine populations. J. Antimicrob. Chemother..

[36] Kadlec K., Schwarz S. (2009). Identification of a novel trimethoprim resistance gene, *dfrK*, in a methicillin-resistant *Staphylococcus aureus* ST398 strain and its physical linkage to the tetracycline resistance gene *tet*(L). Antimicrob. Agents Chemother..

[37] Monclús L., Lopez-Bejar M., De la Puente J., Covaci A., Jaspers V.L.B. (2019). Can variability in corticosterone levels be related to POPs and OPEs in feathers from nestling cinereous vultures (*Aegypius monachus*)?. Sci. Total Environ..

[38] Gómez P., Lozano C., Camacho M.C., Lima-Barbero J.F., Hernández J.M., Zarazaga M., Höfle Ú., Torres C. (2016). Detection of MRSA ST3061-t843-*mecC* and ST398-t011-*mecA* in white stork nestlings exposed to human residues. J. Antimicrob. Chemother..

[39] O’Neill A.J., McLaws F., Kahlmeter G., Henriksen A.S., Chopra I. (2007). Genetic basis of resistance to fusidic acid in staphylococci. Antimicrob. Agents Chemother..

[40] Novick R.P., Ram G. (2017). Staphylococcal pathogenicity islands—Movers and shakers in the genomic firmament. Curr. Opin. Microbiol..

[41] Martins K.B., Faccioli P.Y., Bonesso M.F., Fernandes S., Oliveira A.A., Dantas A., Zafalon L.F., Maria de Lourdes R.S. (2017). Characteristics of resistance and virulence factors in different species of coagulase-negative staphylococci isolated from milk of healthy sheep and animals with subclinical mastitis. J. Dairy Sci..

[42] Saber H., Jasni A.S., Jamaluddin T.Z.M.T., Ibrahim R. (2017). A review of staphylococcal cassette chromosome *mec* (SCC*mec*) types in coagulase-negative staphylococci (CoNS) species. Malays. J. Med. Sci..

