# Supplementary material for: Frequency and Characterization of Antimicrobial Resistance and Virulence Genes of Coagulase-Negative Staphylococci from Wild Birds in Spain. Detection of tst-Carrying S. sciuri Isolates
Source: Microorganisms. 2020 Aug 29;8(9):1317. doi: 10.3390/microorganisms8091317 (PMC7564563; doi:10.3390/microorganisms8091317)
Supplement: Supplementary file 1 [file microorganisms-08-01317-s001.pdf]

Frequency and Characterization of Antimicrobial Resistance and Virulence Genes of Coagulase-Negative Staphylococci from Wild Birds in Spain. Detection of *tst*-carrying *S. sciuri* Isolates.

Laura Ruiz-Ripa, Paula Gómez, Carla Andrea Alonso, María Cruz Camacho, Yolanda Ramiro, Javier de la Puente, Rosa Fernández-Fernández, Miguel Ángel Quevedo, Juan Manuel Blanco, Gerardo Báguena, Myriam Zarazaga, Ursula Höfle, Carmen Torres*

Table S1. Primer pairs used for the characterization of CoNS recovered from wild birds.

Target gene	Primer Sequence (5′-3′)	Amplicon size (bp)	Reference
optrA	F: AGGTGGTCAGCGAACTAA<br>R: ATCA ACTGTTCCCATTCA	1395	[1]
poxtA	F: TCAATGCAGAGCAGGAAGCA<br>R: GGTGGATTTACCGACACCGT	791	[2]
cfr	F: TGAAGTATAAAGCAGGTT GGGAGTCA<br>R: ACCATATAATTGACCACA AGCAGC	746	[3]
***cfr*(B)**	F: TGAGCATATACGAGTAACCTCAAGA<br>R: CGCAAGCAGCGTCTATATCA	293	[4]
***cfr*(D)**	F: AGAAGTCGCAACAAGTGAGGA<br>R: GCAACTGCATGAGTCAAAGAA	595	[5]
fexA	F: GTACTTGTAGGTGCAATTACGGCTGA<br>R: CGCATCTGAGTAGGACATAGCGTC	1272	[6]
fexB	F: TTCCCACTATTGGTGAAAGGAT<br>R: GCAATTCCCTTTTATGGACGTT	816	[7]
$\boldsymbol{cat}_{\mathbf{pC194}}$	F: CGACTTTTAGTATAACCACAGA<br>R: GCCAGTCATTAGGCCTAT	579	[8]
$\boldsymbol{cat}_{\mathbf{pC221}}$	F: ATTTATGCAATTATGGAAGTTG<br>R: TGAAGCATGGTAACCATCAC	434	

cat_{pC223}	F: GAATCAAATGCTAGTTTTAACTC	283	
	R: ACATGGTAACCATCACATAC		
blaZ	F: CAGTTCACATGCCAAAGAG	772	[8]
	R: TACACTCTTGGCGGTTTC		
mecA	F: GGGATCATAGCGTCATTATTC	527	[9]
	R: AACGATTGTGACACGATAGCC		
mecB	F: TTAACATATACACCCGCTTG	527	[10]
	R: TAAAGTTCATTAGGCACCTCC		
mecC	F: GCTCCTAATGCTAATGCA	304	[11]
	R: TAAGCAATAATGACTACC		
***erm*(A)**	F: TCTAAAAAGCATGTAAAAGAA	645	
	R: CTTCGATAGTTTATTAATATTAG		
***erm*(B)**	F: GAAAAGTACTCAACCAAATA	639	[12]
	R: AGTAACGGTACTTAAATTGTTTA		
***erm*(C)**	F: TCAAAACATAATATAGATAAA	642	
	R: GCAAATATTGTTTAAATCGTCAAT		
***erm*(T)**	F: CCGCCATTGAAATAGATCCT	200	[13]
	R : TTCTGTAGCTGTGCTTTCAAAAA		
***erm*(43)**	F: TACAGC AGATGATAACATTG	609	[14]
	R: GTTGTTTCGATATTTTA TTTAAG		
***msr*(A)**	F: GGCACAATAAGAGTGTTTAA AGG	399	[15]

	R: AAGTTATATCATGAATAGATTGTCCTGTT		
***mph*(C)**	F: ATGACTCGACATAATGAAAT<br>R: CTACTCTTTCATACCTAACTC	900	[8]
***sal*(A)**	F: CTATTAATCGATGAACCAACAAACC<br>R: TTGATTTACCTGTACCATTTCTGC	610	[16]
***lnu*(A)**	F: GGTGGCTGGGGGGTAGATGTATTAACTGG<br>R: GCTTCTTTTGAAATACATGGTATTTTTCGATC	322	[17]
***lnu*(B)**	F: CCTACCTATTGTTTGTGGAA<br>R: ATAACGTTACTCTCCTATTC	944	[18]
***lsa*(B)**	F: TGCCGAAGCCATGTACCGTCC<br>R: CGGTTAGACCAACCAGCCGAACG	396	[13]
***vga*(A)**	F: AGTGGTGGTGAAGTAACACG<br>R: GGTTCAATACTCAATCGACTGAG	1264	[19]
***aac*(6')-Ie-*aph*(2'')-Ia**	F: CCAAGAGCAATAAGGGCATA<br>R: CACTATCATAACCACTACCG	220	[20]
***ant*(6)-Ia**	F: ACTGGCTTAATCAATTTGGG<br>R: GCCTTTCCGCCACCTCACCG	597	[21]
str	F: TATTGCTCTCGAGGGTTC<br>R: CTTTCTATATCCATTCATCTC	646	[8]
***ant*(4')-Ia**	F: GCAAGGACCGACAACATTTC<br>R: TGGCACAGATGGTCATAACC	165	[20]

***tet*(K)**	F: TTAGGTGAAGGGTTAGGTCC<br>R: GCAAACTCATTCCAGAAGCA	697	[22]
***tet*(L)**	F: CATTTGGTCTTATTGGATCG<br>R: ATTACACTTCCGATTTCGG	456	
***tet*(M)**	F: GTTAAATAGTGTTCTTGGAG<br>R: CTAAGATATGGCTCTAACAA	576	
fusB	F: CTATAATGATATTAATGAGATTTTTGG<br>R: TTTTTACATATTGACCATCCGAATTGG	431	[23]
fusC	F: TTAAAGAAAAAGATATTGATATCTCGG<br>R: TTTACAGAATCCTTTTACTTTATTTGG	332	
fusD	F: AATTCGGTCAACGATCCC<br>R: TTTTTACATATTGACCATCCGAATTGG	431	[24]
dfrA	F: CCTTGGCACTTACCAAATG<br>R: CTGAAGATTCGACTTCCC	374	[8]
dfrD	F: TTCTTTAATTGTTGCGATGG<br>R: TTAACGAATTCTCTCATATATATG	582	
dfrG	F: TCGGAAGAGCCTTACCTGACAGAA<br>R: CCCTTTTTGGGCAAATACCTCATTCCA	323	[13]
dfrK	F: GAGAATCCCAGAGGATTGGG<br>R: CAAGAAGCTTTTCGCTCATAAA	423	
**Amino acid changes**			

**GyrA**	F: ATGAGYGTTATCGTKCWCGTGC<br>R: CCATWGARCCAAAGTTACCTTG	261	[25]
**GrlA**	F: AATACRYAYGATAARAATTTCCG<br>R: GTYGTRTCATCATAGTTTGG	289	
**SCC*mec*-typing**			
ccrA1-ccrB	F: AACCTATATCATCAATCAGTACGT<br>R: ATTGCCTTGATAATAGCCITCT	695	[26]
ccrA2-ccrB	F: TAAAGGCATCAATGCACAAACACT<br>R: ATTGCCTTGATAATAGCCITCT	937	
ccrA3-ccrB	F: AGCTCAAAAGCAAGCAATAGAAT<br>R: ATTGCCTTGATAATAGCCITCT	1791	
ccrA4-ccrB4	F: TTTGAATGCCCTCCATGAATAAAAT<br>R: AGAAAAGATAGAAGTTCGAAAGA	1287	
ccrC	F: CACTTAATCCATGTACACAG<br>R: TTAGGAAATCTTGATGGCAA	336	[27]
mecA-mecI	F: CATAACTTCCCATTCTGCAGATG<br>R: ATATACCAAACCCGACAACTACA	1963	[26]
mecA-IS1272	F: ATGCTTAATGATAGCATCCGAATG<br>R: ATATACCAAACCCGACAACTACA	2827	
mecA-IS431	F: TGAGGTTATTCAGATATTTCGATGT<br>R: ATATACCAAACCCGACAACTACA	804	

**Virulence genes**			
***lukS*/*F*-PV**	F: ATCATTAGGTAAAATGTCTGGACATGATCCA<br>R: GCATCAAGTGTATTGGATAGCAAAAGC	443	[17]
tst	F: TTCACTATTTGTAAAAGTGTCAGACCCACT<br>R: TACTAATGAATTTTTTTATCGTAAGCCCTT	180	[28]
eta	F: ACTGTAGGAGCTAGTGCATTTGT<br>R: TGGATACTTTTGTCTATCTTTTTCATCAAC	616	
etb	F: CAGATAAAGAGCTTTATACACACATTAC<br>R: AGTGAACTTATCTTTCTATTGAAAAACACTC	1553	
etd	F: AACTATCATGTATCAAGG<br>R: CAGAATTTCCCGACTCAG	402	[29]
**Immune Evasion Cluster (IEC)**			
scn	F: AGCACAAGCTTGCCAACATCG<br>R: TTAATATTTACTTTTTAGTGC	257	[30]

Table S2. Antimicrobial resistance phenotype and genotype, and virulence gene content of the 173 CoNS recovered from free-ranging birds.

Isolate	Species	Origin	Sampling year	Antimicrobial resistance phenotype[a]	Antimicrobial resistance genotype	Virulence gene content
**C9166**	*S. sciuri*	Magpie	2015	PEN-FOX-CLI[I]-FUS	*mecA*, *sal*(A)	-
**C9167**	*S. sciuri*	Magpie	2015	CLI-TET-FUS	*lnu*(A), *sal*(A), *tet*(K)	-
**C9174**	*S. sciuri*	Red kite	2016	CLI[I]	*sal*(A)	-
**C9175**	*S. sciuri*	Red kite	2016	CLI[I]	*lnu*(A), *sal*(A)	-
**C9177**	*S. sciuri*	Red kite	2016	CLI-FUS	*lnu*(A), *sal*(A)	-
**C9179**	*S. sciuri*	Red kite	2016	CLI[I]	*sal*(A)	-
**C9181**	*S. sciuri*	Red kite	2016	CLI-FUS	*sal*(A)	-
**C9183**	*S. sciuri*	Red kite	2016	CLI[I]	*sal*(A)	-
**C9185**	*S. sciuri*	Red kite	2016	CLI[I]-FUS	*sal*(A)	-
**C9186**	*S. sciuri*	Red kite	2016	PEN-FOX-CLI-FUS	*mecA*, *sal*(A)	-
**C9188**	*S. sciuri*	Red kite	2016	CLI[I]	*lnu*(A), *sal*(A)	-
**C9191**	*S. sciuri*	Red kite	2016	CLI[I]	*sal*(A)	-
**C9193**	*S. sciuri*	Red kite	2016	CLI-FUS	*lnu*(A), *sal*(A)	-
**C9194**	*S. sciuri*	Red kite	2016	CLI-FUS	*lnu*(A), *sal*(A)	-
**C9197**	*S. sciuri*	Red kite	2016	CLI-TET-FUS	*sal*(A), *tet*(K)	-
**C9198**	*S. sciuri*	Red kite	2016	PEN-FOX-CLI-TET-FUS	*mecA*, *sal*(A), *tet*(K)	-

**C9200**	*S. sciuri*	Red kite	2016	CLI-TET-FUS	*lnu*(A), *sal*(A), *tet*(K), *tet*(M)	-
**C9202**	*S. sciuri*	Red kite	2016	CLI-FUS	*lnu*(A), *sal*(A)	-
**C9203**	*S. sciuri*	Red kite	2016	CLI[I]	*sal*(A)	-
**C9206**	*S. sciuri*	Red kite	2015	CLI[I]-FUS	*sal*(A)	-
**C9207**	*S. sciuri*	Red kite	2015	PEN-FOX-CLI[I]-FUS	*mecA*, *sal*(A)	-
**C9208**	*S. sciuri*	Red kite	2015	CLI-FUS	*lnu*(A), *sal*(A)	-
**C9211**	*S. sciuri*	Red kite	2015	ERY-CLI-FUS	*erm*(C), *sal*(A)	-
**C9212**	*S. sciuri*	Red kite	2015	PEN-FOX-CLI[I]-FUS	*mecA*, *sal*(A)	-
**C9213**	*S. sciuri*	Red kite	2015	CLI[I]-FUS	*sal*(A)	-
**C9214**	*S. sciuri*	Red kite	2015	CLI-FUS	*sal*(A)	-
**C9215**	*S. sciuri*	Red kite	2015	PEN-FOX-CLI[I]-FUS	*mecA*, *sal*(A)	-
**C9216**	*S. sciuri*	Black-headed gull	2015	CLI[I]-FUS	*sal*(A)	-
**C9217**	*S. sciuri*	Black-headed gull	2015	PEN-FOX-CLI[I]-FUS	*mecA*, *sal*(A)	-
**C9218**	*S. sciuri*	Black-headed gull	2015	PEN-CLI-FUS	*blaZ*, *sal*(A)	-
**C9219**	*S. sciuri*	Black-headed gull	2015	PEN-ERY-CLI[I]-FUS	*erm*(C), *lnu*(A), *sal*(A)	-
**C9220**	*S. sciuri*	Black-headed gull	2015	PEN-FOX-CLI-FUS	*mecA*, *sal*(A)	-
**C9222**	*S. sciuri*	Cinereous vulture	2015	PEN-FOX-ERY-CLI-STR-TET-FUS	*mecA*, *erm*(B), *lnu*(A), *sal*(A), *str*, *tet*(M)	-
**C9223**	*S. sciuri*	Cinereous vulture	2015	CLI-FUS	*sal*(A)	-
**C9224**	*S. sciuri*	Cinereous vulture	2015	PEN-CLI-FUS	*lnu*(A), *sal*(A)	-

**C9225**	*S. sciuri*	Cinereous vulture	2015	PEN-CLI-FUS	*sal*(A)	-
**C9226**	*S. sciuri*	Cinereous vulture	2015	PEN-CLI- FUS	*blaZ, sal*(A)	-
**C9227**	*S. sciuri*	Cinereous vulture	2015	PEN-FOX-CLI-FUS	*mecA, sal*(A)	-
**C9229**	*S. sciuri*	Cinereous vulture	2015	PEN-CLI-FUS	*sal*(A)	-
**C9230**	*S. sciuri*	Cinereous vulture	2015	PEN-CLI-CHL-FUS	*lnu*(A), *sal*(A), *fexA*	-
**C9231**	*S. sciuri*	Cinereous vulture	2015	CLI-FUS	*sal*(A)	-
**C9232**	*S. sciuri*	Cinereous vulture	2015	PEN-CLI-FUS	*sal*(A)	-
**C9233**	*S. sciuri*	Cinereous vulture	2015	PEN-FOX-CLI^{I}-FUS	*mecA, sal*(A)	-
**C9234**	*S. sciuri*	Cinereous vulture	2015	PEN-CLI-STR-FUS	*sal*(A), *str*	-
**C9235**	*S. sciuri*	Cinereous vulture	2015	PEN-CLI-FUS	*sal*(A)	-
**C9236**	*S. sciuri*	Cinereous vulture	2015	PEN-CLI-CHL-FUS	*sal*(A), *fexA*	-
**C9238**	*S. sciuri*	Cinereous vulture	2015	PEN-FOX-CLI-FUS	*mecA, sal*(A)	-
**C9239**	*S. sciuri*	Cinereous vulture	2015	CLI^{I}-FUS	*sal*(A)	-
**C9240**	*S. sciuri*	Cinereous vulture	2015	PEN-FOX-CLI^{I}-FUS	*mecA, sal*(A)	-
**C9244**	*S. sciuri*	Cinereous vulture	2015	PEN-FOX-CLI^{I}-FUS	*mecA, sal*(A)	-
**C9245**	*S. sciuri*	Cinereous vulture	2015	CLI^{I}-FUS	*sal*(A)	-
**C9246**	*S. sciuri*	Cinereous vulture	2015	CLI^{I}-FUS	*lnu*(A), *sal*(A)	-
**C9248**	*S. sciuri*	Cinereous vulture	2015	PEN-CLI-FUS	*sal*(A)	-
**C9249**	*S. sciuri*	Cinereous vulture	2015	PEN-FOX-CLI-STR-FUS	*mecA, sal*(A), *str*	-
**C9252**	*S. sciuri*	Cinereous vulture	2015	PEN-CLI^{I}-FUS	*sal*(A)	-
**C9256**	*S. sciuri*	Cinereous vulture	2015	ERY-CLI-FUS	*erm*(B), *erm*(C), *sal*(A)	-

**C9258**	*S. sciuri*	Cinereous vulture	2015	ERY-CLI-TOB-CIP-FUS	*erm*(B), *msr*(A), *sal*(A), *ant*(4′)-Ia	-
**C9260**	*S. sciuri*	Cinereous vulture	2015	PEN-FOX-CLI-FUS	*mecA*, *sal*(A)	-
**C9262**	*S. sciuri*	Cinereous vulture	2015	ERY-CLI^{I}-FUS	*erm*(C), *sal*(A)	-
**C9263**	*S. sciuri*	Cinereous vulture	2015	PEN-FOX-CLI-TOB-FUS	*mecA*, *blaZ*, *sal*(A), *ant*(4′)-Ia	-
**C9264**	*S. sciuri*	Cinereous vulture	2015	CLI^{I}-FUS	*sal*(A)	-
**C9266**	*S. sciuri*	Bearded vulture	2015	PEN-CLI^{I}-FUS	*sal*(A)	-
**C9270**	*S. sciuri*	Bearded vulture	2016	PEN-FOX-CLI-FUS	*mecA*, *sal*(A)	-
**C9274**	*S. sciuri*	Western marsh harrier	2016	CLI^{I}	*sal*(A)	-
**C9462**	*S. sciuri*	Magpie	2016	PEN-FOX-CLI-STR-FUS	*mecA*, *sal*(A), *str*	-
**C9464**	*S. sciuri*	Magpie	2016	PEN-ERY-CLI-FUS	*erm*(B), *erm*(C), *sal*(A)	-
**C9465**	*S. sciuri*	Magpie	2016	CLI-FUS	*lnu*(A), *sal*(A)	-
**C9466**	*S. sciuri*	Magpie	2016	PEN-CLI-FUS	*blaZ*, *lnu*(A), *sal*(A)	-
**C9467**	*S. sciuri*	Magpie	2016	PEN-CLI-GEN-TOB-FUS	*sal*(A), *aac*(6′)-Ie-*aph*(2′′)-Ia	-
**C9468**	*S. sciuri*	Magpie	2016	CLI^{I}-FUS	*sal*(A)	-
**C9470**	*S. sciuri*	Magpie	2016	CLI^{I}-FUS	*sal*(A)	-
**C9471**	*S. sciuri*	Magpie	2016	CLI-FUS	*sal*(A)	-
**C9472**	*S. sciuri*	Magpie	2016	CLI^{I}-FUS	*sal*(A)	-
**C9473**	*S. sciuri*	Magpie	2016	CLI^{I}-FUS	*sal*(A)	-
**C9474**	*S. sciuri*	Magpie	2016	PEN-CLI^{I}-FUS	*sal*(A)	-

**C9475**	*S. sciuri*	Magpie	2016	PEN-CLI-FUS	*blaZ*, *sal*(A)	-
**C9476**	*S. sciuri*	Magpie	2016	PEN-FOX-ERY-CLI-FUS	*mecA*, *erm*(C), *lnu*(A), *sal*(A)	-
**C9477**	*S. sciuri*	Magpie	2016	PEN-FOX-CLI-STR-FUS	*mecA*, *sal*(A), *str*	-
**C9478**	*S. sciuri*	Cinereous vulture	2016	CLI^{I}-FUS	*sal*(A)	-
**C9479**	*S. sciuri*	Cinereous vulture	2016	ERY-CLI-STR-FUS	*erm*(B), *sal*(A), *str*	-
**C9480**	*S. sciuri*	Cinereous vulture	2016	CLI^{I}-STR-FUS	*sal*(A), *str*	-
**C9481**	*S. sciuri*	Cinereous vulture	2016	PEN-CLI^{I}-FUS	*sal*(A)	-
**C9483**	*S. sciuri*	Cinereous vulture	2016	CLI^{I}-FUS	*sal*(A)	-
**C9484**	*S. sciuri*	Cinereous vulture	2016	CLI-STR-TET-FUS	*lnu*(A), *sal*(A), *str*, *tet*(L)	-
**C9487**	*S. sciuri*	Cinereous vulture	2016	CLI^{I}-FUS	*lnu*(A), *sal*(A)	-
**C9488**	*S. sciuri*	Cinereous vulture	2016	CLI-FUS	*lnu*(A), *sal*(A)	-
**C9489**	*S. sciuri*	Cinereous vulture	2016	CLI^{I}-FUS	*sal*(A)	-
**C9491**	*S. sciuri*	Cinereous vulture	2016	PEN-CLI^{I}-FUS	*sal*(A)	-
**C9493**	*S. sciuri*	Cinereous vulture	2016	PEN-CLI-FUS	*blaZ*, *sal*(A)	-
**C9494**	*S. sciuri*	Cinereous vulture	2016	PEN-CLI^{I}-FUS	*sal*(A)	-
**C9497**	*S. sciuri*	Cinereous vulture	2016	CLI-FUS	*sal*(A)	-
**C9498**	*S. sciuri*	Cinereous vulture	2016	CLI-FUS	*sal*(A)	-
**C9499**	*S. sciuri*	Cinereous vulture	2016	PEN-ERY-CLI-FUS	*erm*(C), *sal*(A)	-
**C9501**	*S. sciuri*	Cinereous vulture	2016	CLI-FUS	*sal*(A)	*tst*
**C9502**	*S. sciuri*	Cinereous vulture	2016	CLI^{I}-TET-FUS	*sal*(A), *tet*(K)	-

**C9503**	*S. sciuri*	Cinereous vulture	2016	PEN-ERY-CLI-FUS	*erm*(C), *sal*(A)	*tst*
**C9505**	*S. sciuri*	Cinereous vulture	2016	CLI-GEN-TOB-FUS	*lsa*(B), sal(A), *aac*(6′)-Ie-*aph*(2′′)-Ia	-
**C9506**	*S. sciuri*	Cinereous vulture	2016	PEN-FOX-CLI^{I}-TOB-FUS	*mecA*, *sal*(A), *ant*(4′)-Ia	-
**C9509**	*S. sciuri*	Cinereous vulture	2016	PEN-CLI-FUS	*sal*(A)	-
**C9512**	*S. sciuri*	Cinereous vulture	2016	CLI^{I}-FUS	*sal*(A)	-
**C9513**	*S. sciuri*	Cinereous vulture	2016	CLI^{I}	*mecA*, *sal*(A)	-
**C9514**	*S. sciuri*	Cinereous vulture	2016	CLI^{I}-GEN-TOB-FUS	*sal*(A), *aac*(6′)-Ie-*aph*(2′′)-Ia,	-
**C9515**	*S. sciuri*	Cinereous vulture	2016	PEN-FOX-CLI^{I}-STR-FUS	*mecA*, *sal*(A), *str*	-
**C9516**	*S. sciuri*	Cinereous vulture	2016	CLI-FUS	*sal*(A)	-
**C9517**	*S. sciuri*	Northern bald ibis	2016	CLI^{I}-FUS	*mecA*, *sal*(A)	-
**C9518**	*S. sciuri*	Northern bald ibis	2016	PEN-CLI-FUS	*blaZ*, *sal*(A)	-
**C9519**	*S. sciuri*	Northern bald ibis	2016	PEN-FOX-CLI^{I}-FUS	*mecA*, *sal*(A)	-
**C9520**	*S. sciuri*	Northern bald ibis	2016	PEN-FOX-CLI-FUS	*mecA*, *sal*(A)	-
**C9521**	*S. sciuri*	Northern bald ibis	2016	PEN-FOX-CLI^{I}-FUS	*mecA*, *sal*(A)	-
**C9522**	*S. sciuri*	Northern bald ibis	2016	PEN-FOX-CLI-FUS	*mecA*, *sal*(A)	-
**C9523**	*S. sciuri*	Northern bald ibis	2016	CLI^{I}-FUS	*sal*(A)	-
**C9525**	*S. sciuri*	Northern bald ibis	2016	PEN-FOX-CLI-FUS	*mecA*, *sal*(A)	-
**C9526**	*S. sciuri*	Northern bald ibis	2016	CLI^{I}-FUS	*sal*(A)	-
**C9527**	*S. sciuri*	Northern bald ibis	2016	PEN-CLI^{I}-TET-FUS	*sal*(A), *tet*(K)	-

**C9528**	*S. sciuri*	Northern bald ibis	2016	CLI^{I}-FUS	*mecA*, *sal*(A)	-
**C9529**	*S. sciuri*	Egyptian vulture	2016	CLI	*sal*(A)	-
**C9530**	*S. sciuri*	Egyptian vulture	2016	CLI-FUS	*sal*(A)	-
**C9533**	*S. sciuri*	European honey buzzard	2016	CLI-FUS	*sal*(A)	-
**C9173**	*S. lentus*	Red kite	2016	ERY-CLI^{In}-TET-FUS	*erm*(B), *erm*(43), *mph*(C), *lnu*(A), *tet*(M)	-
**C9176**	*S. lentus*	Red kite	2016	CLI-FUS	*lnu*(A), *lsa*(B)	-
**C9180**	*S. lentus*	Red kite	2016	PEN-FOX-ERY-CLI-TET-CIP^{b}-FUS	*mecA*, *erm*(B), *erm*(43), *mph*(C), *vga*(A), *tet*(M)	-
**C9182**	*S. lentus*	Red kite	2016	PEN-FOX-ERY-CLI-STR-TET-CIP^{b}-FUS	*mecA*, *erm*(B), *erm*(43), *mph*(C), *str*, *tet*(M)	-
**C9184**	*S. lentus*	Red kite	2016	CLI^{I}-TET-FUS	*lnu*(A), *tet*(K)	-
**C9187**	*S. lentus*	Red kite	2016	CLI-STR-TET-FUS	*lnu*(A), *vga*(A), *str*, *tet*(K)	-
**C9189**	*S. lentus*	Red kite	2016	PEN-ERY-CLI-FUS	*blaZ*, *erm*(C)	-
**C9190**	*S. lentus*	Red kite	2016	ERY-CLI-TET-CIP^{b}-FUS	*erm*(B), *mph*(C), *tet*(M)	-
**C9192**	*S. lentus*	Red kite	2016	ERY-CLI-TET-FUS	*erm*(B), *erm*(43), *mph*(C), *lnu*(A), *tet*(K)	-
**C9195**	*S. lentus*	Red kite	2016	PEN-FOX-ERY-CLI-CIP^{b}-FUS	*mecA*, *blaZ*, *erm*(C), *mph*(C)	-
**C9196**	*S. lentus*	Red kite	2016	CLI-TET-FUS	*lnu*(A), *tet*(K)	-

**C9199**	*S. lentus*	Red kite	2016	PEN-FOX-CLI^{I}-TET-FUS	*mecA*, *tet*(K)	-
**C9201**	*S. lentus*	Red kite	2016	ERY-CLI-FUS	*erm*(B), *erm*(43), *mph*(C)	-
**C9204**	*S. lentus*	Red kite	2016	ERY-CLI-TET	*erm*(C), *tet*(K)	-
**C9205**	*S. lentus*	Red kite	2016	CLI^{I}-TET-FUS	*lnu*(A), *tet*(K)	-
**C9209**	*S. lentus*	Red kite	2015	CLI^{I}-TET-FUS	*lnu*(A), *tet*(K)	-
**C9210**	*S. lentus*	Red kite	2015	PEN-FOX-ERY-CLI-TET-CIP[b]-FUS	*mecA*, *blaZ*, *erm*(C), *erm*(43), *mph*(C), *tet*(K)	-
**C9250**	*S. lentus*	Cinereous vulture	2015	ERY-CLI	*erm*(B), *erm*(43), *mph*(C)	-
**C9251**	*S. lentus*	Cinereous vulture	2015	ERY^{I}-CLI^{I}	*erm*(B), *mph*(C)	-
**C9254**	*S. lentus*	Cinereous vulture	2015	CLI-TET-FUS	*lnu*(A), *tet*(K)	-
**C9265**	*S. lentus*	Bearded vulture	2015	CLI-TOB-STR-TET-SXT	*lnu*(A), *ant*(4')-Ia, *str*, *tet*(K), *tet*(L), *tet*(M), *dfrK*	-
**C9272**	*S. lentus*	Western marsh harrier	2016	PEN-CLI^{I}-FUS	*lnu*(A)	-
**C9482**	*S. lentus*	Cinereous vulture	2016	FUS		-
**C9510**	*S. lentus*	Cinereous vulture	2016	ERY-CLI-FUS	*erm*(B)	-
**C9511**	*S. lentus*	Cinereous vulture	2016	CLI-FUS	*lnu*(A)	-
**C9268**	*S. fleurettii*	Bearded vulture	2015	PEN-FOX-CLI^{I}-STR-FUS	*mecA*, *str*	-
**C9485**	*S. fleurettii*	Cinereous vulture	2016	PEN-FOX-CLI^{I} -FUS	*mecA*	-
**C9486**	*S. fleurettii*	Cinereous vulture	2016	PEN-FOX-CLI^{I} -STR-FUS	*mecA*, *str*	-
**C9492**	*S. fleurettii*	Cinereous vulture	2016	PEN-FOX-CLI^{I} -STR-FUS	*mecA*, *str*	-
**C9495**	*S. fleurettii*	Cinereous vulture	2016	PEN-FOX-FUS	*mecA*	-

**C9504**	*S. fleurettii*	Cinereous vulture	2016	PEN-CLI^{I}-FUS	*mecA*	-
**C9507**	*S. fleurettii*	Cinereous vulture	2016	PEN-CLI-FUS	*mecA*, *lnu*(A)	-
**C9168**	*S. vitulinus*	Magpie	2016	FUS		-
**C9171**	*S. vitulinus*	Magpie	2016	PEN-FOX-STR-FUS	*mecA*, *str*	-
**C9172**	*S. vitulinus*	Magpie	2016	CLI		-
**C9273**	*S. vitulinus*	Western marsh harrier	2016	CLI^{I}-FUS		-
**C9463**	*S. vitulinus*	Magpie	2016	PEN-FOX-FUS	*mecA*	-
**C9490**	*S. vitulinus*	Cinereous vulture	2016	STR-GEN-TOB-FUS	*aac*(6′)-Ie-*aph*(2′′)-Ia, *str*	-
**C9163**	*S. epidermidis*	Magpie	2015	PEN-ERY-CLI-FUS	*blaZ*, *erm*(C), *lnu*(A), *fusB*	-
**C9170**	*S. epidermidis*	Magpie	2016	PEN-FOX-CLI^{I}-FUS	*mecA*, *lnu*(A), *vga*(A)	-
**C9271**	*S. epidermidis*	Bearded vulture	2016	PEN-FOX-ERY-CLI^{I}-TET	*mecA*, *blaZ*, *erm*(C), *mph*(C), *lnu*(A), *tet*(K)	-
**C9524**	*S. epidermidis*	Northern bald ibis	2016	PEN-ERY-CLI	*blaZ*, *erm*(C), *mph*(C)	-
**C9259**	*S. kloosii*	Cinereous vulture		PEN-CLI-TET-FUS	*blaZ*, *lnu*(A), *tet*(K)	-
**C9532**	*S. kloosii*	European honey buzzard	2016	PEN-FUS	*blaZ*	-
**C9534**	*S. kloosii*	European honey buzzard	2016	PEN-FUS	*blaZ*	-
**C9267**	*S. schleiferi* subsp. *schleiferi*	Bearded vulture	2015	CLI-STR-TET	*lnu*(A), *str*, *tet*(K)	-
**C9269**	*S. schleiferi* subsp. *schleiferi*	Bearded vulture	2015	CLI^{I}-STR-TET	*lnu*(A), *str*, *tet*(K)	-
**C9508**	*S. schleiferi* subsp. *schleiferi*	Cinereous vulture		PEN-STR-TET	*blaZ*, *str*, *tet*(K)	-

**C9255**	*S. xylosus*	Cinereous vulture		PEN	*blaZ*	-
**C9275**	*S. xylosus*	Western marsh harrier	2016	PEN-ERY-CLI-TET-FUS	*erm*(B), *mph*(C), *lnu*(A), *tet*(K)	-
**C9500**	*S. xylosus*	Cinereous vulture	2016	PEN-CLI-STR-CIP-FUS	*str*	-
**C9496**	*S. saprophyticus*	Cinereous vulture	2016	PEN	*blaZ*, *fusD*	-
**C9531**	*S. saprophyticus*	European honey buzzard	2016	GEN-TOB-FUS	*aac*(6′)-Ie-*aph*(2′′)-Ia, *fusD*	-
**C9164**	*S. capitis*	Magpie	2015	Susceptible		-
**C9469**	*S. succinus*	Magpie	2016	PEN	*blaZ*	-

[a]PEN, penicillin; FOX, cefoxitin; ERY, erythromycin; CLI, clindamycin; GEN, gentamicin; TOB, tobramycin; STR, streptomycin; TET, tetracycline; CIP, ciprofloxacin; CHL, chloramphenicol; FUS, fusidic acid; SXT, trimethoprim-sulfamethoxazole; I, intermediate resistance; In, inducible resistance.

[b]These isolates showed amino acid changes in GyrA (S84L) and GrlA proteins (D84E and M89L).

Table S3. SCC*mec*-typing of the 47 CoNS isolates from wild birds that carried the *mecA* gene.

Isolate	*ccr*	*mec* complex	SCC*mec*
***S. sciuri* C9166**	-	-	-
***S. sciuri* C9186**	-	-	-
***S. sciuri* C9198**	-	A	-
***S. sciuri* C9207**	*ccrA3-ccrB3*	A	III
***S. sciuri* C9212**	*ccrA2-ccrB2, ccrA3-ccrB3*	A	-
***S. sciuri* C9215**	*ccrA3-ccrB3*	A	III
***S. sciuri* C9217**	-	-	-
***S. sciuri* C9220**	-	-	-
***S. sciuri* C9222**	*ccrA2-ccrB2, ccrA3-ccrB3*	A	-
***S. sciuri* C9227**	-	-	-
***S. sciuri* C9233**	*ccrA3-ccrB3*	A	III
***S. sciuri* C9238**	-	-	-
***S. sciuri* C9240**	-	-	-
***S. sciuri* C9244**	*ccrA3-ccrB3*	A	III
***S. sciuri* C9249**	-	-	-
***S. sciuri* C9260**	-	-	-
***S. sciuri* C9263**	*ccrA3-ccrB3*	A	III
***S. sciuri* C9270**	*ccrA3-ccrB3*	A	III
***S. sciuri* C9462**	-	-	

***S. sciuri* C9476**	*ccrA1-ccrB1, ccrA3-ccrB3*	-	
***S. sciuri* C9477**	*ccrA1-ccrB1, ccrA3-ccrB3*	-	
***S. sciuri* C9506**	-	-	
S. sciuri* C9513	-	-	-
***S. sciuri* C9515**	*ccrA3-ccrB3*	A	III
S. sciuri* C9517	-	-	-
***S. sciuri* C9519**	-	-	
***S. sciuri* C9520**	*ccrA3-ccrB3*	A	III
***S. sciuri* C9521**	-	-	
***S. sciuri* C9522**	*ccrA3-ccrB3*	A	III
***S. sciuri* C9525**	*ccrA3-ccrB3*	A	III
S. sciuri* C9528	-	-	
***S. lentus* C9180**	*ccrC*	-	
***S. lentus* C9182**	*ccrA3-ccrB3*	A	III
***S. lentus* C9195**	-	-	
***S. lentus* C9199**	*ccrA3-ccrB3*	A	III
***S. lentus* C9210**	-	A	
***S. epidermidis* C9170**	*ccrC*	-	-
***S. epidermidis* C9271**	*ccrA2-ccrB2*	B	IV
***S. vitulinus* C9171**	-	-	-
***S. vitulinus* C9463**	-	-	-

S. fleurettii **C9268**	-	-	-
S. fleurettii **C9485**	-	A	-
S. fleurettii **C9486**	-	A	-
S. fleurettii **C9492**	-	A	-
S. fleurettii **C9495**	-	A	-
S. fleurettii **C9504***	-	A	-
S. fleurettii **C9507***	-	A	-

Isolates with an asterisk are those that did not exhibit a methicillin resistance phenotype.

References

1. Wang, Y.; Lv, Y.; Cai, J.; Schwarz, S.; Cui, L.; Hu, Z.; Zhang, R.; Li, J.; Zhao, Q.; He, T.; et al. A novel gene, *optrA*, that confers transferable resistance to oxazolidinones and phenicols and its presence in *Enterococcus faecalis* and *Enterococcus faecium* of human and animal origin. *J. Antimicrob. Chemother.* **2015**, *70*, 2182–2190, doi:10.1093/jac/dkv116.
2. Ruiz-Ripa, L.; Feßler, A.T.; Hanke, D.; Sanz, S.; Olarte, C.; Mama, O.M.; Eichhorn, I.; Schwarz, S.; Torres, C. Coagulase-negative staphylococci carrying *cfr* and PVL genes, and MRSA/MSSA-CC398 in the swine farm environment. *Vet. Microbiol.* **2020**, *243*, 108631, doi:10.1016/j.vetmic.2020.108631.
3. Kehrenberg, C.; Schwarz, S. Distribution of florfenicol resistance genes *fexA* and *cfr* among chloramphenicol-resistant *Staphylococcus* isolates. *Antimicrob. Agents Chemother.* **2006**, *50*, 1156–1163, doi:10.1128/AAC.50.4.1156-1163.2006.
4. Lee, S.M.; Huh, H.J.; Song, D.J.; Shim, H.J.; Park, K.S.; Kang, C.I.; Ki, C.S.; Lee, N.Y. Resistance mechanisms of linezolid-nonsusceptible enterococci in Korea: Low rate of 23s rRNA mutations in *Enterococcus faecium*. *J. Med. Microbiol.* **2017**, *66*, 1730–1735, doi:10.1099/jmm.0.000637.
5. Ruiz-Ripa, L.; Feßler, A.T.; Hanke, D.; Eichhorn, I.; Azcona-Gutiérrez, M.; Pérez-Moreno, M.O.; Seral, C.; Aspiroz, C.; Alonso, C.A.; Torres, L.; et al. Mechanisms of Linezolid Resistance Among Enterococci of Clinical Origin in Spain — Detection of *optrA* - and *cfr*(D)-Carrying *E. faecalis*. **2020**, doi:10.3390/microorganisms8081155.
6. Kehrenberg, C.; Schwarz, S. Florfenicol-chloramphenicol exporter gene *fexA* is part of the novel transposon Tn*558*. *Antimicrob. Agents Chemother.* **2005**, *49*, 813–815, doi:10.1128/AAC.49.2.813-815.2005.
7. Liu, H.; Wang, Y.; Wu, C.; Schwarz, S.; Shen, Z.; Jeon, B.; Ding, S.; Zhang, Q.; Shen, J. A novel phenicol exporter gene, *fexB*, found in enterococci of animal origin. *J. Antimicrob. Chemother.* **2012**, *67*, 322–325, doi:10.1093/jac/dkr481.
8. Schnellmann, C.; Gerber, V.; Rossano, A.; Jaquier, V.; Panchaud, Y.; Doherr, M.G.; Thomann, A.; Straub, R.; Perreten, V. Presence of new *mecA* and *mph*(C) variants conferring antibiotic resistance in *Staphylococcus* spp. isolated from the skin of horses before and after clinic admission. *J. Clin. Microbiol.* **2006**, *44*, 4444–4454, doi:10.1128/JCM.00868-06.
9. Poulsen, A.B.; Skov, R.; Pallesen, L. V. Detection of methicillin resistance in coagulase-negative staphylococci and in staphylococci directly from simulated blood cultures using the EVIGENE MRSA Detection Kit. *J. Antimicrob. Chemother.* **2003**, *51*, 419–421, doi:10.1093/jac/dkg084.
10. Becker, K.; van Alen, S.; Idelevich, E.A.; Schleimer, N.; Seggewiß, J.; Mellmann, A.; Kaspar, U.; Peters, G. Plasmid-encoded transferable *mecB*-mediated methicillin resistance in *Staphylococcus aureus*. *Emerg. Infect. Dis.* **2018**, *24*, 242–248, doi:10.3201/eid2402.171074.
11. Cuny, C.; Layer, F.; Strommenger, B.; Witte, W. Rare occurrence of methicillin-resistant *Staphylococcus aureus* CC130 with a novel

mecA homologue in humans in Germany. *PLoS One* **2011**, *6*, doi:10.1371/journal.pone.0024360.
12. Sutcliffe, J.; Grebe, T.; Tait-Kamradt, A.; Wondrack, L. Detection of erythromycin-resistant determinants by PCR. *Antimicrob. Agents Chemother.* **1996**, *40*, 2562–2566, doi:10.1128/aac.40.11.2562.
13. Gómez-Sanz, E.; Torres, C.; Lozano, C.; Fernández-Pérez, R.; Aspiroz, C.; Ruiz-Larrea, F.; Zarazaga, M. Detection, molecular characterization, and clonal diversity of methicillin-resistant *Staphylococcus aureus* CC398 and CC97 in Spanish slaughter pigs of different age groups. *Foodborne Pathog. Dis.* **2010**, *7*, 1269–1277, doi:10.1089/fpd.2010.0610.
14. Schwendener, S.; Perreten, V. New MLS B resistance gene *erm*(43) in *Staphylococcus lentus*. *Antimicrob. Agents Chemother.* **2012**, *56*, 4746–4752, doi:10.1128/AAC.00627-12.
15. Wondrack, L.; Massa, M.; Yang, B. V.; Sutcliffe, J. Clinical strain of *Staphylococcus aureus* inactivates and causes efflux of macrolides. *Antimicrob. Agents Chemother.* **1996**, *40*, 992–998, doi:10.1128/aac.40.4.992.
16. Hot, C.; Berthet, N.; Chesneau, O. Characterization of *sal*(A), a Novel Gene Responsible for Lincosamide and Streptogramin a resistance in *Staphylococcus sciuri*. *Antimicrob. Agents Chemother.* **2014**, *58*, 3335–3341, doi:10.1128/AAC.02797-13.
17. Lina, G.; Piémont, Y.; Godail-Gamot, F.; Bes, M.; Peter, M.O.; Gauduchon, V.; Vandenesch, F.; Etienne, J. Involvement of Panton-Valentine leukocidin-producing *Staphylococcus aureus* in primary skin infections and pneumonia. *Clin. Infect. Dis.* **1999**, *29*, 1128–1132, doi:10.1086/313461.
18. Bozdogan, B.; Berrezouga, L.; Kou, M.S.; Yurek, D.A.; Farley, K.A.; Stockman, B.J.; Leclercq, R. A new resistance gene, *linB*, conferring resistance to lincosamides by nucleotidylation in *Enterococcus faecium* HM1025. *Antimicrob. Agents Chemother.* **1999**, *43*, 925–929, doi:10.1128/aac.43.4.925.
19. Lozano, C.; Aspiroz, C.; Rezusta, A.; Gómez-Sanz, E.; Simon, C.; Gómez, P.; Ortega, C.; Revillo, M.J.; Zarazaga, M.; Torres, C. Identification of novel *vga*(A)-carrying plasmids and a Tn*5406*-like transposon in meticillin-resistant *Staphylococcus aureus* and S*taphylococcus epidermidis* of human and animal origin. *Int. J. Antimicrob. Agents* **2012**, *40*, 306–312, doi:10.1016/j.ijantimicag.2012.06.009.
20. van de Klundert, J.; Vliegenthart, J. PCR detection of genes coding for aminoglycoside- modifying enzymes. In *Diagnostic Molecular Microbiology. Principles and Applications*; 1993; pp. 547–552.
21. Clark, N.C.; Olsvik, Ø.; Swenson, J.M.; Spiegel, C.A.; Tenover, F.C. Detection of a streptomycin/spectinomycin adenylyltransferase gene (*aadA*) in *Enterococcus faecalis*. *Antimicrob. Agents Chemother.* **1999**, *43*, 157–160, doi:10.1128/aac.43.1.157.
22. Aarestrup, F.M.; Agerso, Y.; Gerner-Smidt, P.; Madsen, M.; Jensen, L.B. Comparison of antimicrobial resistance phenotypes and resistance genes in *Enterococcus faecalis* and *Enterococcus faecium* from humans in the community, broilers, and pigs in Denmark. *Diagn. Microbiol. Infect. Dis.* **2000**, *37*, 127–137, doi:10.1016/S0732-8893(00)00130-9.
23. Mclaws, F.; Chopra, I.; O'Neill, A.J. High prevalence of resistance to fusidic acid in clinical isolates of *Staphylococcus epidermidis*. *J.*

Antimicrob. Chemother. **2008**, *61*, 1040–1043, doi:10.1093/jac/dkn071.
24. Chen, H.J.; Hung, W.C.; Tseng, S.P.; Tsai, J.C.; Hsueh, P.R.; Teng, L.J. Fusidic acid resistance determinants in *Staphylococcus aureus* clinical isolates. *Antimicrob. Agents Chemother.* **2010**, *54*, 4985–4991, doi:10.1128/AAC.00523-10.
25. Intorre, L.; Vanni, M.; Di Bello, D.; Pretti, C.; Meucci, V.; Tognetti, R.; Soldani, G.; Cardini, G.; Jousson, O. Antimicrobial susceptibility and mechanism of resistance to fluoroquinolones in *Staphylococcus intermedius* and *Staphylococcus schleiferi*. *J. Vet. Pharmacol. Ther.* **2007**, *30*, 464–469, doi:10.1111/j.1365-2885.2007.00896.x.
26. Kondo, Y.; Ito, T.; Ma, X.X.; Watanabe, S.; Kreiswirth, B.N.; Etienne, J.; Hiramatsu, K. Combination of multiplex PCRs for staphylococcal cassette chromosome *mec* type assignment: Rapid identification system for *mec*, *ccr*, and major differences in junkyard regions. *Antimicrob. Agents Chemother.* **2007**, *51*, 264–274, doi:10.1128/AAC.00165-06.
27. Witte, W.; Strommenger, B.; Stanek, C.; Cuny, C. Methicillin-resistant *Staphylococcus aureus* ST398 in humans and animals, central Europe. *Emerg. Infect. Dis.* **2007**, *13*, 255–258, doi:10.3201/eid1302.060924.
28. Jarraud, S.; Mougel, C.; Thioulouse, J.; Lina, G.; Meugnier, H.; Forey, F.; Nesme, X.; Etienne, J.; Vandenesch, F. Relationships between *Staphylococcus aureus* genetic background, virulence factors, *agr* groups (alleles), and human disease. *Infect. Immun.* **2002**, *70*, 631–641, doi:10.1128/IAI.70.2.631.
29. Yamaguchi, T.; Nishifuji, K.; Sasaki, M.; Fudaba, Y.; Aepfelbacher Amagai, M.; Takata, T.; Ohara, M.; Komatsuzawa, H.; Aepfelbacher Amagai, M.; Yamaguchi, T.; et al. Identification of the *Staphylococcus aureus etd* pathogenicity island which encodes a novel exfoliative toxin, ETD, and EDIN-B. *Infect. Immun.* **2002**, *70*, 5835–5845, doi:10.1128/IAI.70.10.5835.
30. Van Wamel, W.J.B.; Rooijakkers, S.H.M.; Kessel, K.P.M. Van; Strijp, J. a G. Van; Ruyken, M. The Innate Immune Modulators Staphylococcal Complement Inhibitor and Chemotaxis Inhibitory Protein of *Staphylococcus aureus* are located on beta-hemolysin-converting bacteriophages. *J. Bacteriol.* **2006**, *188*, 1310–1315, doi:10.1128/JB.188.4.1310.
